# Toxin Production by *Alternaria alternata* in Black Spot Disease of *Chrysanthemum morifolium* ‘Fubai’: Accumulation of Altenuene and Tenuazonic Acid in Flowers

**DOI:** 10.3390/toxins17040181

**Published:** 2025-04-05

**Authors:** Qingling Zhan, Lina Liu, Wenjie Li, Jingshan Lu, Jiafu Jiang, Fadi Chen, Ye Liu, Zhiyong Guan

**Affiliations:** National Key Laboratory of Crop Genetics & Germplasm Enhancement, Key Laboratory of Landscape Design, Ministry of Agriculture & Rural Affairs, Nanjing Agricultural University, Nanjing 210095, China; 2022204014@stu.njau.edu.cn (Q.Z.); t2024101@njau.edu.cn (W.L.); jingshan@njau.edu.cn (J.L.); jiangjiafu@njau.edu.cn (J.J.); chenfd@njau.edu.cn (F.C.)

**Keywords:** *Chrysanthemum morifolium* ‘Fubai’, black spot disease, *Alternaria alternata*, altenuene (ALT), tenuazonic acid (TeA), floral contamination, food safety

## Abstract

*Alternaria* species produce diverse secondary metabolites that act as critical virulence factors during plant pathogenesis. In cultivation areas of *Chrysanthemum morifolium* ‘Fubai’—a key cultivar for herbal tea—black spot disease caused by *A. alternata* manifests as necrotic leaf lesions progressing to wilting. Despite this disease’s economic impact, information on its associated toxins is limited, and the types of toxins produced by the black spot pathogen of *Chrysanthemum morifolium* ‘Fubai’ in particular remain unclear. Furthermore, whether toxins are present in the flowers when the leaves show symptoms is uncertain, but their inflorescence is not visibly infected. Using two previously characterized *A. alternata* strains (F16/F20) isolated from ‘Fubai’ in earlier studies, we demonstrated the concomitant production of altenuene (ALT) and tenuazonic acid (TeA) in both strains, with strain-specific yield variations (F20 TeA: 342.16 µg/mL vs. F16: 21.84 µg/mL; ALT: 0.28 µg/mL vs. 0.90 µg/mL). Time-course monitoring revealed coordinated accumulation of both toxins in inoculated petals, reaching 18.07 μg/g ALT and 2.59 µg/g TeA by day 9. Notably, these two toxins were detected only in flower samples from black spot-infected plants, and their concentrations correlated closely with disease severity in the leaves. Moreover, although the inflorescences did not display symptoms, both fresh and dried flowers retained detectable toxin levels. We established a technical system for the extraction and quantitative detection of the toxins ALT and TeA produced by the black spot pathogen in tea chrysanthemum. This work provides the first confirmation of ALT/TeA co-contamination in *Chrysanthemum morifolium* ‘Fubai’, revealing substantial dietary exposure risks through tea consumption. Our findings suggest that, from a food safety risk reduction perspective, integrated management strategies should be developed to minimize toxin contamination in tea chrysanthemum, including improved disease prevention measures and potential regulatory considerations.

## 1. Introduction

*Chrysanthemum morifolium* ‘Fubai’ is a perennial cultivar of the genus *Chrysanthemum* that is cultivated primarily on the southern slopes of the Dabie Mountains in Macheng, Hubei Province, China. It has been granted National Geographical Indication (NGI) status and is recognized as a protected agricultural commodity, reinforcing its importance as a key medicinal resource in Hubei Province. Along with *Chrysanthemum morifolium* ‘Hang-bai’ and *Chrysanthemum morifolium* ‘Gongju’, it is recognized as one of China’s three major medicinal chrysanthemums and is economically valuable. However, recurrent outbreaks of black spot disease (*Alternaria alternata*) in *Chrysanthemum morifolium* ‘Fubai’ plantations compromise yield stability [1]. Under high humidity and temperature, the pathogen induces necrotic leaf lesions, leading to growth suppression, flower abortion, and systemic plant collapse.

*Alternaria* spp., facultative saprophytes with broad host ranges and epidemic dispersal traits [2,3], produce cell wall-degrading enzymes and *Alternaria* toxins (ATs) during infection [4]. As key virulence factors, ATs not only drive host colonization and disease progression [5] but also pose direct threats to human health due to their mutagenic, genotoxic, carcinogenic, and acutely toxic properties [6,7,8,9]. More than 70 ATs with significant toxicity have been identified from *Alternaria* spp. [10,11,12], with notable metabolites including alternariol (AOH), alternariol methyl ether (AME), altenuene (ALT), tenuazonic acid (TeA), and tentoxin (Ten) [13,14,15,16]. Notably, ATs produced in infected leaves can translocate to surrounding healthy tissues, where they accumulate, exacerbating disease severity. Among these, TeA is particularly potent, inducing severe leaf deterioration and contributing to brown spot formation [17].

Despite extensive studies on AT contamination in fruits [18,19], vegetables [20,21,22], grains [23,24], and edible oils [25], their translocation dynamics in medicinal chrysanthemums—particularly their potential accumulation in asymptomatic floral tissues—remain uncharacterized, highlighting a critical knowledge gap. The European Food Safety Authority (EFSA) has established thresholds of toxicological concern (TTC) values for AOH/AME (2.5 ng/kg body weight/day) and TeA/Ten (1500 ng/kg body weight/day) [26], while TeA is recognized as a hazardous compound by U.S. regulatory agencies. However, two critical questions remain unresolved. Specifically, whether ATs generated by black spot disease in infected leaves translocate to chrysanthemum flowers and whether commercially processed dried flowers retain residual toxins. To address these analytical gaps, we developed a modified QuEChERS (Quick, Easy, Cheap, Effective, Rugged, and Safe) sample preparation technique. This method was coupled with ultra-high-performance liquid chromatography–tandem mass spectrometry (UHPLC-MS/MS) and ultra-performance liquid chromatography (UPLC) for comprehensive AT contamination analysis. This approach allowed us to systematically investigate AT contamination profiles across four critical stages: fungal cultures, inoculated petals, field-infected flowers, and commercially dried products.

## 2. Results

### 2.1. UHPLC-MS/MS Qualitative Detection

**Figure 1:**
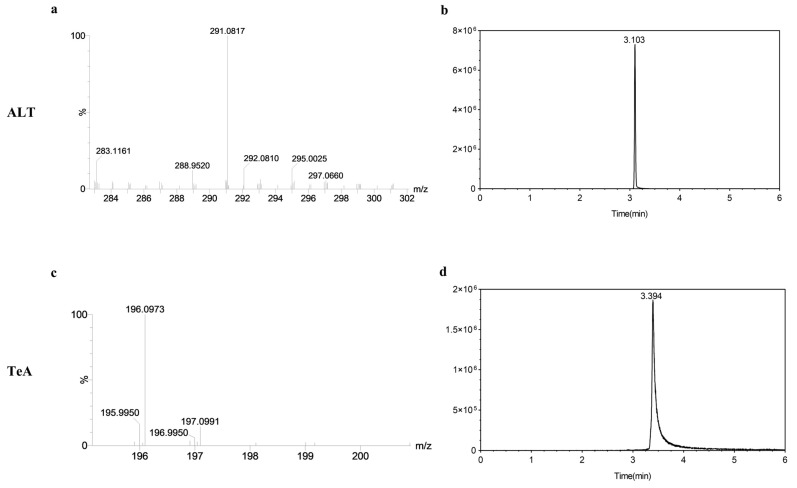
UPLC-MS/MS spectrometry and chromatogram of altenuene (ALT) and tenuazonic acid (TeA). (**a**) UHPLC-MS/MS spectrometry of ALT. (**b**) UHPLC-MS/MS chromatogram of ALT. (**c**) UHPLC-MS/MS spectrometry of TeA. (**d**) UHPLC-MS/MS chromatogram of TeA.

The qualitative analysis of toxin extracts from strains F16 and F20 was conducted following the UHPLC-MS/MS conditions. During the analysis, a comparison of the mass-to-charge ratios of the five toxins revealed that the culture filtrates of the two strains exhibited a pronounced response at the mass-to-charge ratios corresponding to ALT and TeA, with peaks occurring at 3.103 s and 3.394 s, respectively. The mass spectra and chromatograms are presented in Figure 1. No response signals or peaks were observed for AOH, AME, and Ten, indicating that strains F16 and F20 produced only ALT and TeA during the incubation process.

### 2.2. UPLC Quantitative Detection for ATs

We further developed a UPLC analytical method for the quantitative detection of the toxins ALT and TeA based on these measurements. The results demonstrated that the two toxins exhibit excellent linearity within a concentration range of 0.1 to 500 μg/mL, with correlation coefficients (*R*^2^) exceeding 0.995 (Figure S1). The limit of detection (LOD) for ALT was 0.023 μg/mL, while the limit of quantification (LOQ) was 0.077 μg/mL. The LOD of TeA was 0.026 μg/mL, and the LOQ was 0.086 μg/mL (Table 1). The recoveries of the two toxins in the spiked samples ranged from 86.65% to 96.78%, with RSDs < 6.0% (Table 2).

### 2.3. Concentration of ALT and TeA in Culture Filtrates of Strains

**Figure 2:**
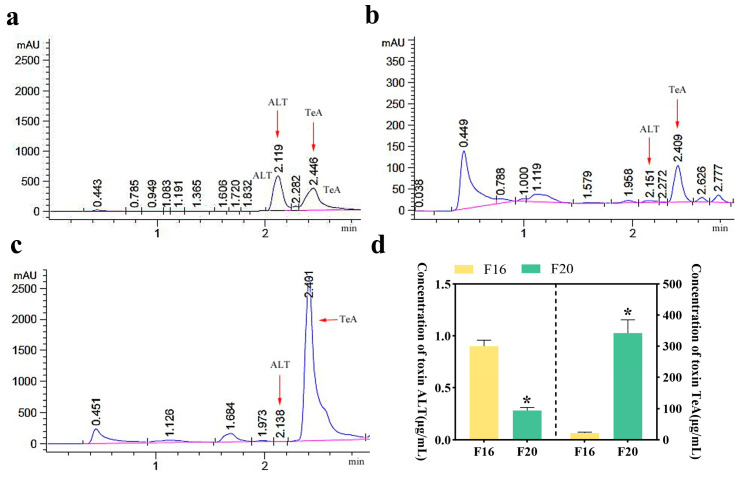
UPLC chromatogram of mixed standard solution of ALT and TeA and toxin production results of strains F16 and F20. (**a**) Concentration of 100 μg/mL of mixed standard solution of ALT and TeA. (**b**) Toxin production results of strain F16. (**c**) Toxin production results of strain F20. (**d**) Concentration of Alternaria toxins in filtrates of *A. alternata* strains cultured in Richard medium for 20 days. All values in the figures are presented as mean ± SD, *n* = 3. * significant differences between strains (F16 vs. F20) determined by Student’s *t*-test (* *p* < 0.05).

The quantitative detection of ATs was conducted using the established UPLC system, with the results illustrated in Figure 2. As observed in Figure 2a, the retention time for ALT in the standard working solution is approximately 2.119 s, and for TeA, it is approximately 2.446 s. The peak retention times of ALT and TeA in strains F16 and F20 were within the acceptable margin of error compared to the control, confirming that both strains produced these toxins during cultivation (a substance is considered identical if its retention time in different concentration gradients of the test samples does not vary by more than 5%).

The toxicity of different *Alternaria* strains was calculated based on the results of the UPLC assay, using the linear equations for the two toxins. As shown in Figure 2d, ALT concentrations ranged from 0.283 to 0.901 μg/mL, whereas TeA levels were markedly higher (21.840–342.157 μg/mL). While the two strains exhibited comparable ALT production, the concentration of TeA produced by F20 was significantly higher than that produced by F16. As the only nitrogen-containing metabolite among known ATs, TeA exhibits the highest virulence and the strongest acute toxic effects [27]. Thus, strain F20 is likely more virulent and suitable for further experimental studies.

### 2.4. Concentration of ALT and TeA in Inoculated Chrysanthemum Petals

**Figure 3:**
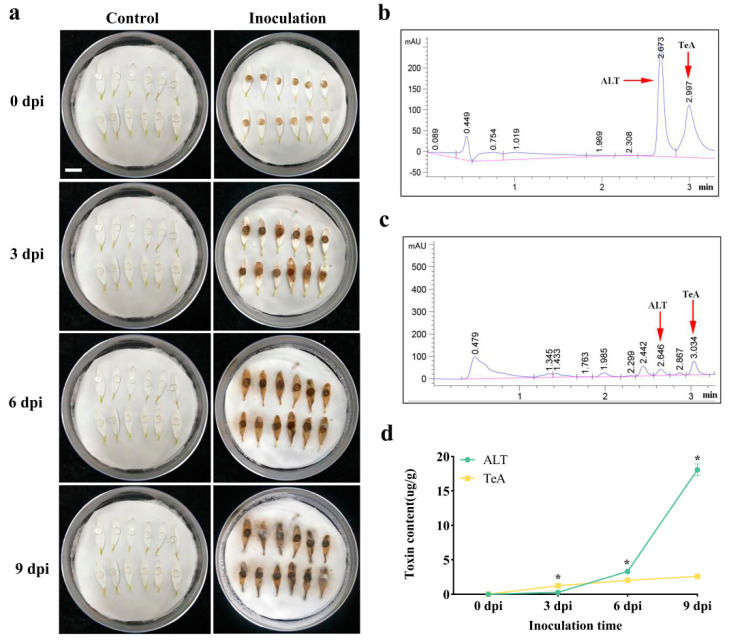
Determination of the contents of *Alternaria* toxins TeA and ALT in *Chrysanthemum morifolium* ‘Fubai’ petals after artificial inoculation. (**a**) Temporal dynamics of ALT and TeA accumulation in tea chrysanthemum petals post-inoculation with *A. alternata*; (**b**) concentration of 10 μg/mL of mixed standard solution of ALT and TeA; (**c**) toxin production results for 3dpi of petals; (**d**) incidence 3, 6, and 9 days after petals were inoculated with *A. alternata* (bar = 1 cm)**.** All values in the figures are presented as mean ± SD, *n* = 3. The asterisks indicate significant differences between the two datasets at the same time point, as determined by *t*-test (* *p* < 0.05).

In view of the above findings, strain F20 was selected to artificially inoculate chrysanthemum petals. Figure 3a shows the development of lesions on the petals following inoculation with the pathogenic fungus. Petal samples were analyzed using the previously described UPLC conditions, and the resulting chromatograms are presented in Figure 3c. As shown in Table 3, the two ATs in the petals exhibited a strong linear relationship within a concentration range of 0.05 to 100 μg/mL, with a high correlation coefficient (*R*^2^ = 0.999).

The two ATs, ALT and TeA, were first detected in chrysanthemum petals on day 3 post-inoculation, with concentrations progressively increasing over time (Figure 3d). By day 9, ALT accumulated to 18.070 ± 0.840 μg/g (mean ± SD), whereas TeA reached 2.593 ± 0.051 μg/g. Initial quantification revealed higher TeA levels compared to ALT on day 3 (TeA: 1.236 μg/g vs. ALT: 0.236 μg/g). However, ALT exhibited exponential growth from day 6 onward, surpassing TeA by 1.63-fold on day 6 and 6.97-fold on day 9 (*p* < 0.05). In contrast, TeA accumulation plateaued after day 6.

### 2.5. Analysis of Toxin Contamination in Field-Collected Flower Samples

The toxins ALT and TeA were not detected in the flowers of healthy *Chrysanthemum morifolium* ‘Fubai’ plants. Table 4 shows that TeA was detected in 100% of the flower samples from susceptible plants. The mean TeA content detected in the flowers of severely infected plants was 1.22 μg/kg, which was 3.8 times higher than that in the flowers of mildly infected plants. ALT was detected in 83% of the fresh flower samples collected from mildly infected plants, with a mean concentration of 1.86 μg/kg. In contrast, a 100% detection rate was observed in the flowers of severely infected plants, where the toxin content averaged 17.15 μg/kg.

In the dried flowers of infected plants, both toxins (TeA and ALT) were detected at 100% frequency. Collectively, the toxin concentrations in the flowers of severely infected plants were significantly higher than those in mildly infected plants. Specifically, the TeA and ALT levels in mildly infected flowers were 1.85 μg/kg and 51.31 μg/kg, respectively. In contrast, severely infected flowers exhibited substantially elevated concentrations, with TeA and ALT reaching 6.16 μg/kg and 95.79 μg/kg, respectively.

**Figure 4:**
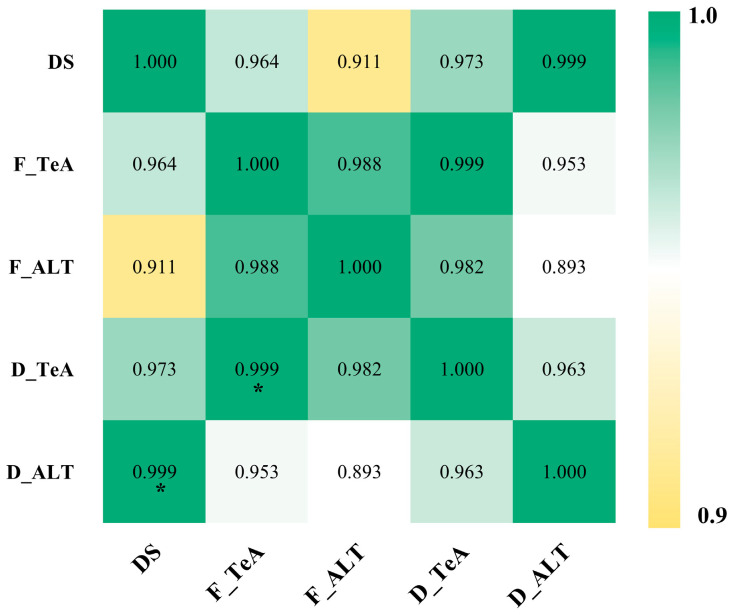
Heatmap of Pearson’s correlations between AT concentrations and severity in chrysanthemum samples. D and F represent fresh and dried samples, respectively. The numbers in the figure indicate correlation coefficients, and * denotes *p* < 0.05.

Pearson’s correlation analysis demonstrated significant positive associations between AT concentrations (ALT/TeA) in chrysanthemum florets and lesion severity indices (Figure 4).

## 3. Discussion

*Alternaria* spp. are destructive plant-pathogenic fungi worldwide. Approximately 500 species of *Alternaria* have been identified, and these species can cause diseases in numerous crops and economically significant plants [28]. These diseases seriously affect the growth, storage, and transportation of these crops, jeopardizing production and leading to substantial economic losses [29]. Mycotoxins are toxic secondary metabolites secreted by pathogenic fungi. These mycotoxins play a crucial role in host infection by disrupting physiological processes [30,31]. Zhang et al. demonstrated that *Alternaria* crude toxin inhibits the growth of the roots and stems of chrysanthemum seedlings, and the degree of inhibition is positively related to the crude toxin concentration [32].

The two *A. alternata* strains F16 and F20, isolated from black spot-diseased leaves of Chrysanthemum morifolium ‘Fubai’, produce the phytotoxins alternariol (ALT) and tenuazonic acid (TeA). Notably, strain F20 exhibits higher TeA production (342.16 μg/mL in culture filtrate) and stronger pathogenicity compared to F16, positioning it as the dominant pathogen during infection [33]. This heightened virulence is attributed to TeA, a chloroplast-targeted toxin that disrupts host defenses by inhibiting photosystem II (PSII) activity [34,35]. Beyond its role in pathogenesis, TeA demonstrates potent herbicidal effects through PSII inhibition, indicating its potential application as a natural herbicide [36,37]. However, its dual functionality as both a virulence factor and agrochemical candidate necessitates rigorous safety evaluations. Despite promising herbicidal applications, TeA poses significant toxicological risks. Acute and chronic toxicity studies reveal adverse effects on mammals, including hepatotoxicity and neurotoxicity [38,39], raising concerns about environmental and food chain contamination. Comprehensive toxicological profiling—encompassing chronic exposure thresholds, ecotoxicological impacts, and degradation pathways—must precede any field application.

While TeA was the predominant toxin in vitro (culture filtrate: 342.16 μg/mL vs. ALT 0.28 μg/mL), ALT accumulated at higher levels in planta (inoculated petals: 18.07 μg/g vs. TeA 2.59 μg/g), suggesting differential toxin translocation mechanisms between culture media and plant tissues. This discrepancy may result from multiple factors, including compositional differences between the culture filtrate and petal tissues, lower humidity during petal inoculation, and the extended incubation period before culture filtrate collection. Additionally, the inoculation period for petal samples was under 20 days, and by the 9th day post-inoculation, the petals had completely browned and decayed.

While *Alternaria* infections primarily target leaves, the flowers of infected plants may also become contaminated. Field tests on chrysanthemum flowers from naturally diseased tea plants showed that flowers from healthy plants were free of toxin contamination. However, flowers from black spot-affected plants (showing symptoms only on leaves) were contaminated, even though they exhibited no visible symptoms such as color or shape changes. Furthermore, as leaf symptoms worsened, the levels of TeA and ALT detected in the flowers increased. Similar findings on the correlation between toxin content and infection severity have been reported in studies on jujube samples [40]. Notably, the two toxins were also detected in dried flowers in this study, suggesting that high-temperature drying during chrysanthemum processing does not effectively degrade these toxins. Most ATs are heat-stable and resistant to degradation during food processing [41]. Thus, high-temperature processing does not mitigate potential drinking safety risks. Currently, due to the lack of data on chronic toxicity and tolerable daily intake, no specific regulatory thresholds for ATs have been established either domestically or internationally, highlighting the urgent need for further risk assessments. In 2022, the European Commission issued recommendations requiring member states to monitor ATs in food, focusing on AOH, AME, and TeA [42]. The potential chronic exposure risks posed by the detected toxin levels in chrysanthemum tea, along with their possible synergistic effects with other toxins, warrant further validation through animal and human risk assessments. Furthermore, establishing stricter regulatory standards could significantly reduce consumer exposure risks.

This study successfully established a rapid detection method for altenuene (ALT) and tenuazonic acid (TeA) produced by *A. alternata* in black spot disease of Chrysanthemum morifolium ‘Fubai’ using UHPLC-MS/MS and UPLC. Compared to UPLC, UHPLC-MS/MS offers higher sensitivity, specificity, and anti-interference capability. Through its multiple-reaction monitoring (MRM) mode, its detection limit can reach the fg-pg level. Additionally, UHPLC-MS/MS can provide molecular ion peaks and fragment ion information, allowing for the direct identification of unknown compounds and enabling simultaneous qualitative detection of multiple components in complex matrices. Therefore, when the types of toxins produced by A. alternata were unknown, we first employed UHPLC-MS/MS for qualitative analysis. On the other hand, UPLC provides higher separation efficiency, faster analysis speed, and stronger system compatibility. By utilizing a 1.7–2.1 μm small-particle short chromatography column, a high-pressure system, and a gradient elution program, UPLC significantly increases the theoretical plate number and reduces the single-analysis time, making it highly suitable for routine quantification, rapid screening, and stability studies. Thus, after identifying ALT and TeA, we used UPLC for the subsequent large-scale quantitative detection of toxin content in culture filtrates and flower samples. Our experimental results clearly demonstrate that the complementary advantages of UHPLC-MS/MS and UPLC can be leveraged to optimize detection efficiency and accuracy, meeting different analytical requirements and significantly improving the overall efficiency of the study.

## 4. Conclusions

In summary, the toxins ALT and TeA produced by the pathogenic fungi of *Chrysanthemum morifolium* ‘Fubai’ can contaminate asymptomatic flowers in the field, with residual toxin levels in flowers showing a strong correlation with the disease severity of the plants. This finding underscores the critical importance of strengthening disease prevention and control throughout the entire growth cycle of tea chrysanthemums to mitigate potential dietary exposure risks posed by toxin residues. Furthermore, the UPLC-based detection system established in this study provides a robust technical framework for the rapid screening of toxin contamination in tea chrysanthemum products.

## 5. Materials and Methods

### 5.1. Materials and Reagents

#### 5.1.1. Fungal Material

*Alternaria alternata* strains F16 and F20 were isolated from symptomatic leaves of Chrysanthemum morifolium ‘Fubai’ in the Chunyang Mountain production area (Macheng, Hubei, China). Pathogenicity was confirmed by fulfilling Koch’s postulates [33]. Species identity was validated through the following analyses:

Morphological characteristics: Both strains formed circular colonies on PDA medium with yellow-brown pigmentation and dense aerial hyphae resembling fluffy mycelia (Figure S2). Conidia were brown to light brown, predominantly obclavate or obpyriform in shape, measuring 24.5–42.5 × 9.5–13.5 μm (*n* = 30 randomly selected mature conidia), and containing 2–6 transverse septa and 1–4 longitudinal septa (Figure S2).

Molecular identification: The strains exhibited 99% sequence identity to *A. alternata* strain KT280010.1 in the NCBI database. Phylogenetic analysis using the neighbor-joining method in MEGA11 placed both strains within the *A. alternata* clade with 100% bootstrap support (Figure S3).

#### 5.1.2. Culture Media and Chemical Reagents

Culture media included Potato Dextrose Agar (PDA), Potato Dextrose Broth (PDB), and modified Richard’s liquid medium (containing 1% KNO₃, 3% sucrose, 0.25% MgSO₄, 0.5% KH₂PO₄, 0.002% FeCl₃, 0.0005% ZnSO₄, and 0.1% yeast extract per liter). Chromatographic-grade formic acid (Aladdin, Beijing, China) and acetonitrile (Merck, Darmstadt, Germany) were used alongside certified AT standards (AOH, AME, ALT, TeA, Ten; purity > 99.0%, Sigma-Aldrich, Housto, TX, USA).

### 5.2. Instruments

#### 5.2.1. Chromatography Systems

Ultra-high-performance liquid chromatography–tandem mass spectrometry (UHPLC-MS/MS): Agilent 1290 system (Agilent Technologies, Santa Clara, CA, USA) equipped with a UV detector and ZORBAX RRHD Eclipse Plus C18 column (2.1 × 50 mm, 1.8 μm).

Ultra-performance liquid chromatography (UPLC): Waters ACQUITY UPLC/Xevo G2-XS QTofX system (Waters Corp., Milford, MA, USA) with a BEH C18 column (2.1 × 100 mm, 1.7 μm).

(Note: UHPLC and UPLC are equivalent techniques; the terminology differs based on vendor conventions.)

#### 5.2.2. Auxiliary Equipment

A vortex mixer (IKA Genius 2, Staufen, Germany), thermostatic shaker (IS-RDD3, Jingqi, Dallas, TX, USA), freeze dryer (LGJ-10FD, Songyuan Huaxing, Beijing, China), and centrifuge (Eppendorf 5430R, Hamburg, Germany) were used.

### 5.3. Sample Collection

#### 5.3.1. Fungal Culture Filtrate

Fungal Culture Filtrate: Eight mycelial disks (5 mm in diameter) of each strain were added to 25 mL of Richard’s liquid medium. After sealing, the cultures were incubated in the dark at 28 °C for 48 h. Subsequently, they were transferred to a 12 h light/12 h dark cycle for further cultivation. After 20 days, the culture medium was filtered using sterile gauze. The filtrate was then centrifuged at 5000 rpm for 10 min, and the supernatant was collected to obtain the culture filtrate containing crude toxins. The filtrate was stored at −20 °C for later use.

#### 5.3.2. Artificially Inoculated Petal Samples

Fresh petals of uniform size and appearance were carefully selected, immersed in 70% ethanol for 5 s, and arranged uniformly in 90 mm Petri dishes. The center of each petal was inoculated with a 5 mm mycelial plug, while Petri dishes containing PDA medium alone served as negative controls. After sealing with Parafilm, all plates were incubated under identical conditions (28 °C, 12 h light/12 h dark cycle). Disease symptoms were monitored daily. At 3, 6, and 9 days post-inoculation (dpi), three biological replicates (independent plants) were collected at each time point, and petals from each replicate were pooled to obtain 1 g (fresh weight) of tissue for toxin extraction and quantification.

#### 5.3.3. Naturally Infected Field Samples

**Figure 5:**
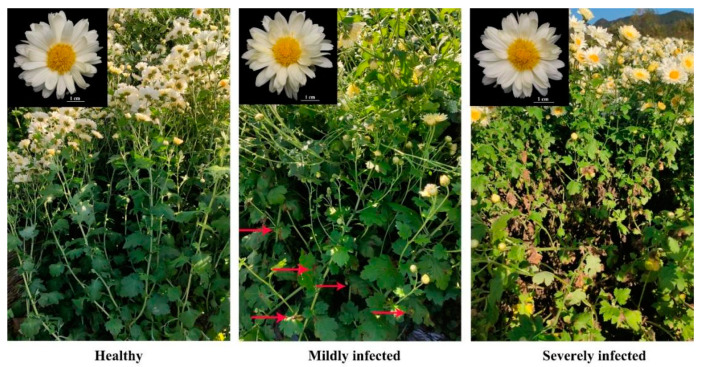
Chrysanthemum for tea with black spot disease in field used for petal toxin determination. Red arrows point to leaves with black spot symptoms.

Chrysanthemum samples were collected from naturally infected plants in the ‘Fubai’ production area. Disease severity (DS) was classified based on the percentage of leaf area covered by lesions: DS = 0% as healthy (Level 0), 0% < DS ≤ 10% as mildly infected (Level 1), and DS ≥ 70% as severely infected (Level 2). Representative symptoms of plants at different disease severity levels are shown in Figure 5. Notably, symptoms were confined to leaves, with the flowers of infected plants showing no visual differences from healthy ones.

Fresh flowers were harvested from the tops of several plants and divided into two portions: one portion was placed in an envelope and thoroughly dried in an oven at 65 °C for dry flower analysis, while the other was used directly for fresh flower analysis (Note: fresh samples were ground with liquid nitrogen, and dried samples were pulverized directly using a laboratory grinder). Both fresh and dried chrysanthemum samples were then ground for further use. Five biological replicates were included for each treatment.

$$\mathrm{Disease}\;\mathrm{severity}\;(\%)=\frac{\sum (lesion\;area\;per\;lear/total\;leaf\;area)}{Total\;number\;of\;investigated\;leaves}\;\times \;100\%$$

### 5.4. Sample Pretreatment

#### 5.4.1. Fungal Culture Filtrate Extraction

According to the QuEChERS sample extraction method with slight modifications [27,43,44], 5 mL of crude toxin solution was transferred into a 50 mL centrifuge tube. Subsequently, 15 mL of acetonitrile acidified with 2% (*v*/*v*) formic acid was added, and the mixture was vortexed at 200 rpm for 30 min. Afterward, 0.5 g of NaCl and 2 g of anhydrous MgSO₄ were added, and the mixture was vigorously shaken for 3 min. The sample was then centrifuged at 7500 rpm for 20 min. The supernatant was collected and subjected to a second centrifugation at 13,000 rpm for 10 min. The resulting supernatant was frozen overnight and then subjected to lyophilization for 12 h to obtain crystalline residues. The crystalline residue was then dissolved in 1 mL of methanol and centrifuged (13,000 rpm, 10 min). The supernatant was filtered through a 0.22 μm organic filter membrane and transferred into a 1.5 mL brown injection vial for UHPLC-MS/MS and UPLC analyses.

#### 5.4.2. Petal Tissue Extraction

The fungi were removed from the petal surface, and 1 g of each sample was weighed into a 5 mL centrifuge tube. Next, 2 mL of sterile water and two sterile stainless steel beads (4 mm diameter) were added, and the sample was ground into a homogenate. The homogenate was then transferred to a 50 mL centrifuge tube, and the subsequent steps were carried out as described in Section 5.4.1.

#### 5.4.3. Fresh and Dried Flower Extraction

Following the accurate weighing of 1 g of fresh flower powder and 0.4 g of dried flower powder, the samples were placed in 50 mL centrifuge tubes, and 15 mL of acidified acetonitrile was added to each. The subsequent treatments were carried out as described in Section 5.4.1, with six replicates set up for each group in view of the large variation in samples in the field.

### 5.5. UPLC-MS/MS Detection Conditions

To identify the toxin profiles of *A. alternata* strains F16 and F20, we employed ultra-high-performance liquid chromatography–tandem mass spectrometry (UHPLC-MS/MS). This method was chosen for its high sensitivity and selectivity, which are critical for detecting trace levels of ATs (e.g., ALT and TeA) in complex biological matrices. UPLC-MS/MS allowed us to perform both qualitative and quantitative analyses, ensuring the accurate identification of the toxin-producing capabilities of the experimental strains.

Chromatographic conditions: The separation was performed using the Agilent 1290 UHPLC system. The mobile phase consisted of 0.1% formic acid in water (mobile phase A) and 0.1% formic acid in acetonitrile (mobile phase B). The flow rate was set at 0.4 mL·min^−1^, with an injection volume of 2.0 μL and a column temperature of 30 °C. Gradient Elution Program (percentage of mobile phase B): 0–0.5 min, 5%; 0.5–15 min; 95%, 16–17 min, 5%. Mass spectrometry conditions: electrospray ionization (ESI) in negative ion mode was used, and all samples were quantified in multiple-reaction monitoring (MRM) mode. Monitored ion pairs (*m*/*z*) for target compounds: ALT: *m*/*z* 291.08 > 258; TeA: *m*/*z* 196.10 > 130; AOH: *m*/*z* 257.2 > 213; AME: *m*/*z* 271.2 > 256.3; TEN: 413.1: *m*/*z* > 140.9. Ionization parameters: capillary voltage: 2.5 kV; sample cone voltage: 40 V; ion source temperature: 120 °C; desolvation gas temperature: 400 °C [45].

### 5.6. UPLC Detection Conditions

Following the identification of ALT and TeA as the primary toxins produced by strains F16 and F20, we optimized an ultra-performance liquid chromatography (UPLC) method to facilitate cost-effective and high-throughput analysis. This approach was tailored for practical applications in agricultural monitoring, where the rapid quantification of large sample volumes is essential. By focusing solely on ALT and TeA (confirmed via UHPLC-MS/MS), we streamlined the chromatographic conditions to enhance efficiency and reduce operational costs.

Chromatographic conditions: The separation was performed using the Waters ACQUITY UPLC/Xevo G2-XS QTofX system. The column temperature was set to 30 °C. The mobile phase consisted of methanol and aqueous solution (2% formic acid). The gradient elution program was as follows: from 0 to 6 min, methanol increased from 40% to 100%, then it was left for 1 min, and then it was returned to 40% and was left for 2 min. The flow rate was 0.3 mL/min, and the injection volume was 4.0 μL. The UV detector was set to an absorption wavelength of 258 nm [46].

### 5.7. Method Validation

The method was validated according to the European guidelines SANTE/11945/2015 [47], with evaluation parameters including selectivity, linearity, limit of detection (LOD), limit of quantification (LOQ), trueness (spiked recovery), and precision (relative standard deviation, RSD).

Preparation of standard solutions: 1 mg of toxin standard was dissolved in 1 mL of methanol to prepare a 1 mg/mL stock solution. The stock solution was stored in sealed amber vials at −20 °C under light-protected conditions. Working standard series were prepared through the serial dilution of the stock solution to achieve final concentrations of 0.05, 0.1, 1, 10, 50, 100, and 500 μg/mL in methanol. All solutions were maintained at −20 °C in light-resistant containers until analysis.

Selectivity and linearity: Selectivity was confirmed by comparing chromatograms of blank matrix and spiked samples, ensuring no interference around the target peaks. Linear fitting was performed with the concentration of the mixed standard solution (X, μg/mL) as the abscissa and the UV absorption peak area (Y, mAU × min) as the ordinate. The linear range covered 0.05–500 μg/mL, with correlation coefficients (*R*^2^) all greater than 0.996.

Limit of detection (LOD) and limit of quantification (LOQ): LOD and LOQ were calculated using the formulas DL = 3 N/K and QL = 10 N/K, respectively, where N represents the baseline noise of the instrument and K is the slope of the standard curve. The LOD and LOQ of each toxin met the requirements for trace detection.

Trueness and precision: To evaluate the trueness and precision of the method, mixed standard solutions of two ATs were added to Richard’s medium samples and petal samples at three different concentration levels (0.1, 1.0, and 10.0 μg/mL). The spiked samples were determined under the optimized pretreatment and analysis conditions. Each concentration level was measured five times, and the relative standard deviations (RSDs) and spiked recovery ranges of the two ATs were calculated.

### 5.8. Data Processing and Analysis

All statistical analyses were performed and graphical presentations were made using GraphPad Prism 10.1.2 for Windows (GraphPad Software Inc., San Diego, CA, USA). Statistical analyses were performed using the independent *t*-test, with significance thresholds defined as * *p* < 0.05. Data are expressed as mean ± SD.

## Figures and Tables

**Table 1:** Detection parameters of ALT and TeA.

Component	Linear Equation	*R* ^2^	Linear Range (μg/mL)	LODs (μg/mL)	LOQs (μg/mL)
ALT	y = 47.722x − 8.022	0.997	0.1–500	0.023	0.077
TeA	y = 58.055x − 826.750	0.996	0.1–500	0.026	0.086

**Table 2:** Recoveries and relative standard deviations of ALT and TeA at different spiked levels.

Component	Spiked (μg/mL)	Average Recovery (%)	RSD (%)
ALT	0.1	85.65	4.3
	1.0	91.37	5.7
	10.0	95.83	3.9
TeA	0.1	88.25	5.0
	1.0	93.41	4.5
	10.0	96.78	3.2

**Table 3:** Detection parameters of ALT and TeA in petals of *Chrysanthemum morifolium* ‘Fubai’.

Component	Linear Equation	Correlation Coefficient (*R*^2)^	Linear Range (μg·g^−1^)	LODs (μg·g^−1^)	LOQs (μg·g^−1^)
ALT	y = 112.07x + 94.335	0.999	0.05–100	0.021	0.172
TeA	y = 197.610x − 149.550	0.999	0.05–100	0.816	0.915

**Table 4:** Concentrations of ALT and TeA in fresh and dried *Chrysanthemum morifolium* ‘Fubai’ flowers used for herbal tea preparation.

Type	Infection Severity		TeA			ALT	
		Detection Rate (%)	Content Range	Average Value (μg.kg^−1^)	Detection Rate (%)	Content Range	Average Value (μg.kg^−1^)
Fresh samples	Healthy	ND	0	0	ND		0
	Mildly infected	100	0.19~0.62	0.32 ± 0.18	80	0~4.91	1.86 ± 2.23
	Severely infected	100	0.9~1.88	1.22 ± 0.38	100	7.89~24.89	17.15 ± 7.18
Dried samples	Healthy	ND	0	0	ND		0
	Mildly infected	80	0.5~3.57	1.95 ± 1.17	100	22.1~77.62	51.31 ± 22.60
	Severely infected	100	4.58~9.04	6.16 ± 1.85	100	80.07~105.5	95.79 ± 10.30

Note: ND refers to not detected. All values in the figures are presented as mean ± SD; *n* = 5.

## Data Availability

The original contributions presented in this study are included in this article and Supplementary Materials. Further inquiries can be directed to the corresponding authors.

## Supplementary Materials

The following supporting information can be downloaded at: https://www.mdpi.com/article/10.3390/toxins17040181/s1, Figure S1: Matrix-matched calibration curve for ALT and TeA; Figure S2: Colony and conidial morphology of the pathogenic fungi isolated from *Chrysanthemum morifolium* ‘Fubai’. Figure S3: phylogenetic analysis of the black spot pathogen isolated from *Chrysanthemum morifolium* ‘Fubai’ based on 18S rDNA-ITS sequences (our previously published study [33]).
