# Supplementary material for: Toxin Production by Alternaria alternata in Black Spot Disease of Chrysanthemum morifolium ‘Fubai’: Accumulation of Altenuene and Tenuazonic Acid in Flowers
Source: Toxins (Basel). 2025 Apr 5;17(4):181. doi: 10.3390/toxins17040181 (PMC12030965; doi:10.3390/toxins17040181)
Supplement: Supplementary file 1 [file toxins-17-00181-s001.zip › toxins-3519108-supplementary.pdf]

toxins MDPI

Supplementary Materials: Toxin Production by *Alternaria alternata* in Black Spot Disease of *Chrysanthemum morifolium* ‘Fubai’: Accumulation of Altenuene and Tenuazonic Acid in Flowers

Qingling Zhan, Lina Liu, Wenjie Li, Jingshan Lu, Jiafu Jiang, Fadi Chen, Ye Liu and Zhiyong Guan

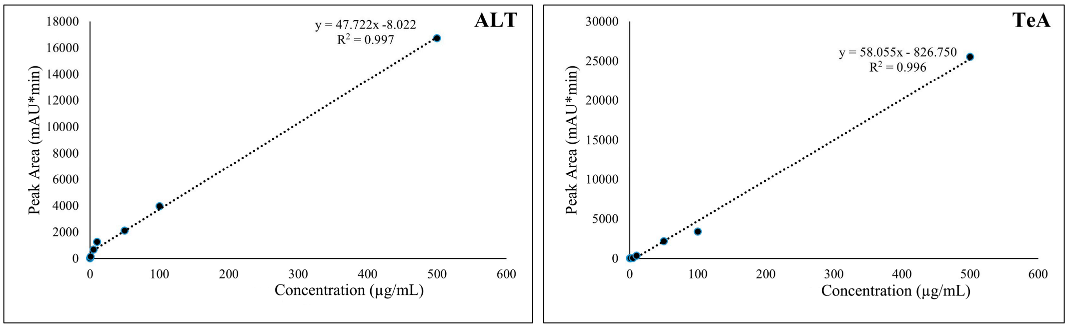

Figure S1. Matrix-matched calibration curve for ALT and TeA.

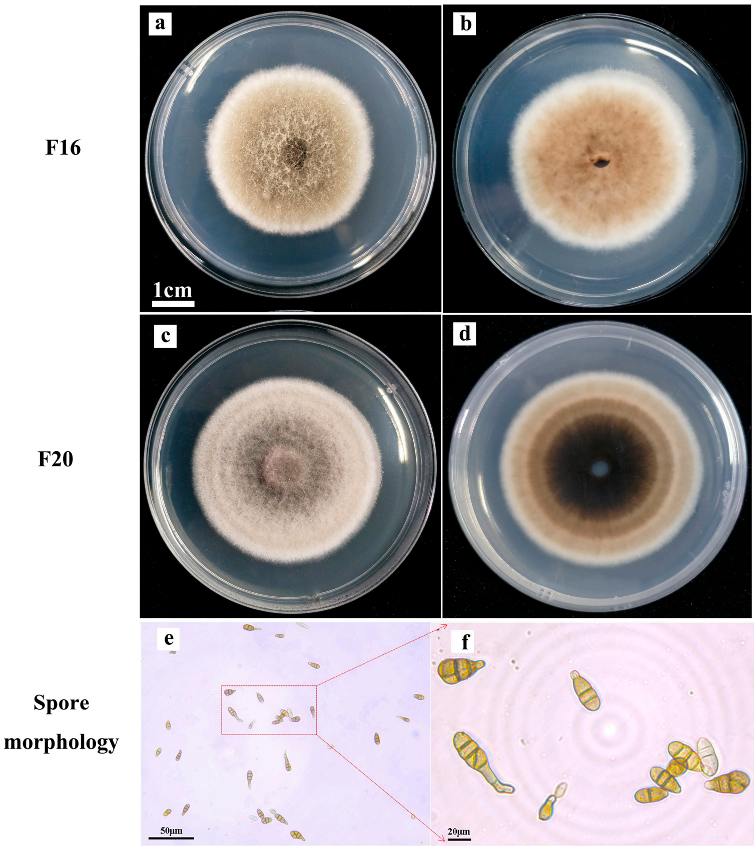

Figure S2. Colony and conidial morphology of the pathogenic fungi isolated from *Chrysanthemum morifolium* 'Fubai'. a: Front view of colony of strain F16; b: Reverse view of colony of strain F20; c: Front view of colony of strain F16; d: Reverse view of colony of

strain F20; e: Conidial morphology under 20× magnification; f: Conidial morphology under 40× magnification (our previously published study [33])

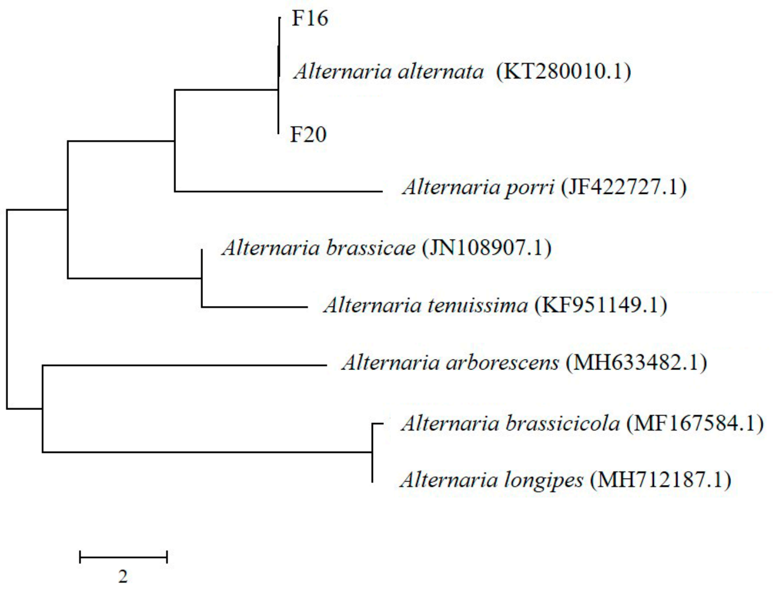

Figure S3. Phylogenetic analysis of the black spot pathogen isolated from *Chrysanthemum morifolium* 'Fubai' based on 18S rDNA-ITS sequences (our previously published study [33]).
